# Cellulose-specific Type B carbohydrate binding modules: understanding oligomeric and non-crystalline substrate recognition mechanisms

**DOI:** 10.1186/s13068-018-1321-7

**Published:** 2018-11-30

**Authors:** Abhishek A. Kognole, Christina M. Payne

**Affiliations:** Department of Chemical and Materials Engineering, University of Kentucky, 177 F Paul Anderson Tower, Lexington, KY 40506 USA

**Keywords:** Amorphous cellulose, β-Sandwich fold, Carbohydrate–aromatic stacking, Free energy perturbation, Multi-modular glycoside hydrolases

## Abstract

**Background:**

Effective enzymatic degradation of crystalline polysaccharides requires a synergistic cocktail of hydrolytic enzymes tailored to the wide-ranging degree of substrate crystallinity. To accomplish this type of targeted carbohydrate recognition, nature produces multi-modular enzymes, having at least one catalytic domain appended to one or more carbohydrate binding modules (CBMs). The Type B CBM categorization encompasses several families (i.e., protein folds) of CBMs that are generally thought to selectively bind oligomeric polysaccharides; however, a subset of cellulose-specific CBM families (17 and 28) appear to bind non-crystalline cellulose more tightly than oligomers and in a manner that discriminates between surface topology.

**Results:**

To provide insight into this unexplained phenomenon, we investigated the molecular-level origins of oligomeric and non-crystalline carbohydrate recognition in cellulose-specific Type B CBMs using molecular dynamics (MD) simulation and free energy calculations. Examining two CBMs from three different families (4, 17, and 28), we describe how protein–ligand dynamics contribute to observed variations in binding affinity of oligomers within the same CBM family. Comparisons across the three CBM families identified factors leading to modified functionality prohibiting competitive binding, despite similarity in sequence and specificity. Using free energy perturbation with Hamiltonian replica exchange MD, we also examined the hypothesis that the open topology of the binding grooves in families 17 and 28 necessitates tight binding of an oligomer, while the more confined family 4 binding groove does not require the same degree of tight binding. Finally, we elucidated the mechanisms of non-crystalline carbohydrate recognition by modeling CBMs complexed with a partially decrystallized cellulose substrate. Molecular simulation provided structural and dynamic data for direct comparison to oligomeric modes of carbohydrate recognition, and umbrella sampling MD was used to determine ligand binding free energy. Comparing both protein–carbohydrate interactions and ligand binding free energies, which were in good agreement with experimental values, we confirmed the hypothesis that family 17 and 28 CBMs bind non-crystalline cellulose and oligomers with different affinities (i.e., high and low).

**Conclusions:**

Our study provides an unprecedented level of insight into the complex solid and soluble carbohydrate substrate recognition mechanisms of Type B CBMs, the findings of which hold considerable promise for enhancing lignocellulosic biomass conversion technology and development of plant cell wall probes.

**Electronic supplementary material:**

The online version of this article (10.1186/s13068-018-1321-7) contains supplementary material, which is available to authorized users.

## Background

Enzymatic hydrolysis of lignocellulosic biomass into fermentable sugars remains a technologically challenging step in the cost-effective production of second-generation biofuels [1]; both enzyme stability and rate of hydrolytic turnover are targets for biotechnological improvement. Substrate composition and crystallinity, as well as efficient substrate recognition by enzymes, are a few of the important factors that determine the yield and rate of enzymatic hydrolysis [2]. Microorganisms often accomplish carbohydrate hydrolysis through the secretion of multi-modular enzymes, wherein carbohydrate binding modules (CBMs) are appended to globular catalytic domains via a linker peptide. CBMs play a valuable role in hydrolysis and recognition processes, as they are responsible for maintaining proximity of the enzyme to the substrate and targeting specific regions of crystallinity [3–5]. These functions serve to enhance catalytic turnover under substrate-limited conditions [6–8]. The ability to target distinct morphological regions of carbohydrate substrates is perhaps the most interesting, yet least understood, function of CBMs and offers great potential in extending the industrial application of CBMs beyond lignocellulosic biomass conversion [9–13]. For example, CBMs show promise as biotechnological tools in applications as wide ranging as affinity chromatography, targeting of functional molecules to cellulosic substrates, cell immobilization technology, enzyme engineering, and fiber modification [4, 5, 8]. As such, there is a critical need to develop understanding of the molecular-level carbohydrate recognition process of CBMs.

CBMs are structurally diverse proteins, binding with many different types of carbohydrate polymorphs and morphologies. To capture this diversity categorically, CBMs have been divided into both families and types based on protein sequence and functional similarity, respectively [3, 14]. Currently, this nomenclature defines function as the ability to target particular substrate crystallinities, as CBMs appear to bind either crystalline or non-crystalline/amorphous and oligomeric substrates. Type A CBMs are specific for crystalline substrates and exhibit a complementary planar binding site lined with aromatic residues [15, 16]. Type B and C CBMs are only subtly different from each other, with both types generally binding oligosaccharides and non-crystalline/amorphous substrates in clefts or grooves. Type B CBMs are capable of binding at any point along the length of the substrate, and Type C CBMs are limited to the end of the oligomer. The underlying protein features enabling this distinction are difficult to define.

The most common protein fold among Type B CBMs is the β-sandwich fold, the proteins of which uniquely recognize not only different kinds of carbohydrates but also varying degrees of polymerization, from the smallest of oligosaccharides to amorphous substrates [3]. This suggests the β-sandwich fold is a versatile architecture that allows relatively minor variations in sequence and, accordingly, chemical properties of the binding cleft/groove to determine carbohydrate binding specificity. Moreover, despite similar substrate specificities and, in some cases, similar measured affinities, some Type B CBMs appear to uncompetitively discriminate between binding sites on variable crystallinity surfaces [16, 17]. Attempts to experimentally characterize non-crystalline/amorphous cellulose have revealed few details of specific structural properties, only that it is cellulose with a decreasing degree of polymerization and crystallinity index [18]. Non-crystalline/amorphous cellulose derived from pretreatment of native crystalline cellulose could be composed of anything from variable-length polysaccharide chains to only partially decrystallized substrate. Thus, the ability to recognize both soluble oligomers and non-crystalline/amorphous cellulose is a key aspect of Type B CBM functionality.

**Fig. 1:**
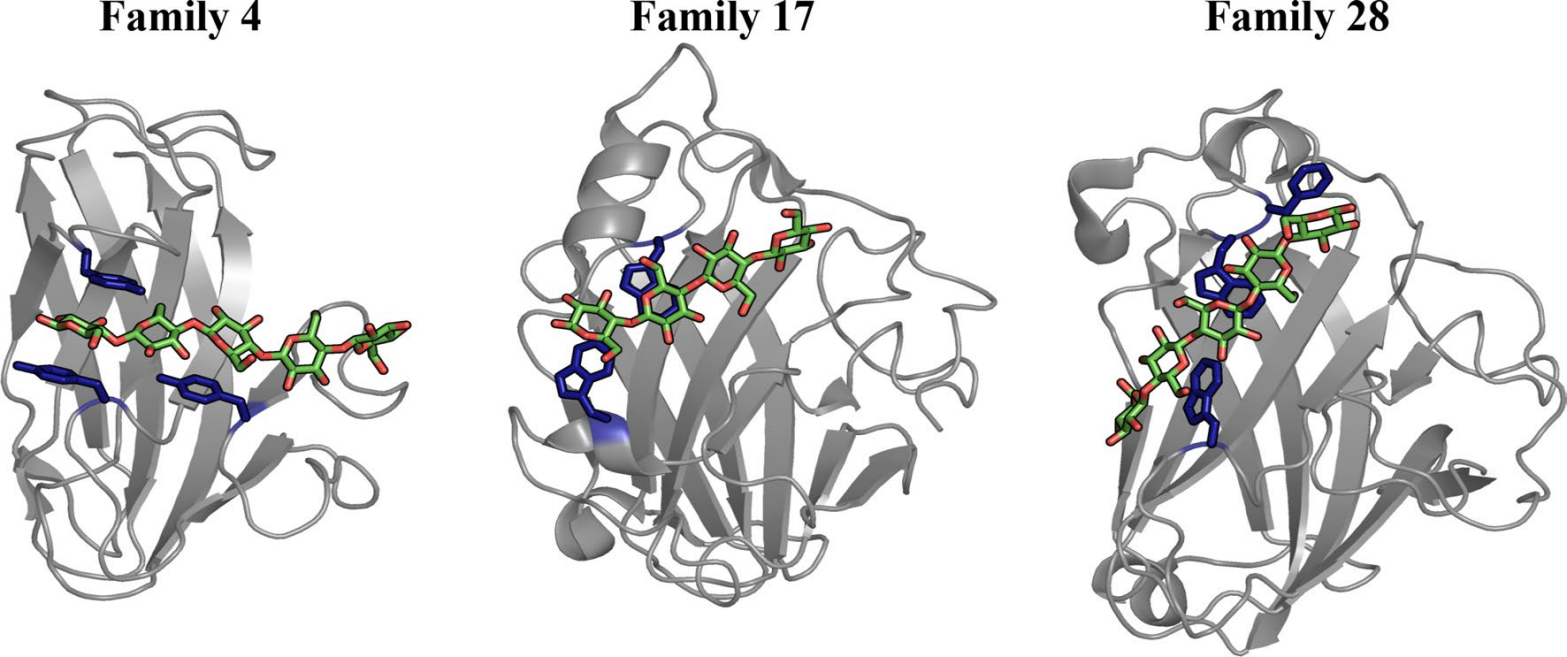
CBMs (cartoon) from families 4, 17, and 28 with bound cello-oligomers (green and red sticks). Binding site aromatic residues are shown in a dark blue stick representation. The structures, *Cellulomonas fimi* CBM4-1, *Clostridium cellulovorans* CBM17, and *Clostridium josui* CBM28, were obtained from crystal structures with PDB IDs 1GU3, 1J84, and 3ACI, respectively. After structural alignment of the β-sandwich proteins, the family 4 and 17 CBM cello-oligomer is bound in the same direction, with the reducing end toward the left of the figure, whereas the family 28 CBM’s cello-oligomer is oriented in the opposite direction

Cellulose-specific Type B CBMs, including those from families 4, 17, and 28, each with the β-sandwich fold (Fig. 1), have been shown to bind both soluble cello-oligomers and non-crystalline cellulose [6–8, 19–23]. Additionally, adsorption isotherms suggest families 17 and 28 individually recognize ‘high’ and ‘low’ affinity binding sites on representative non-crystalline cellulose substrates [13]. These studies also reveal that family 17 and 28 Type B CBMs exhibit higher affinities towards non-crystalline cellulose than toward oligomeric substrates [13, 24]. *Cellulomonas fimi* CBM4–1 and CBM4–2 (*Cf*CBM4–1 and *Cf*CBM4–2, respectively) also appear to bind cello-oligomers bi-directionally, with the reducing end of the pyranose ring at either end of the cleft; there is positive, but limited, evidence of this phenomenon being common among β-sandwich CBMs [11, 25]. Collectively, the data imply that these Type B CBMs are discriminating between the various available binding sites on the non-crystalline carbohydrate surface, but there is not necessarily a directional preference within the binding site.

**Fig. 2:**
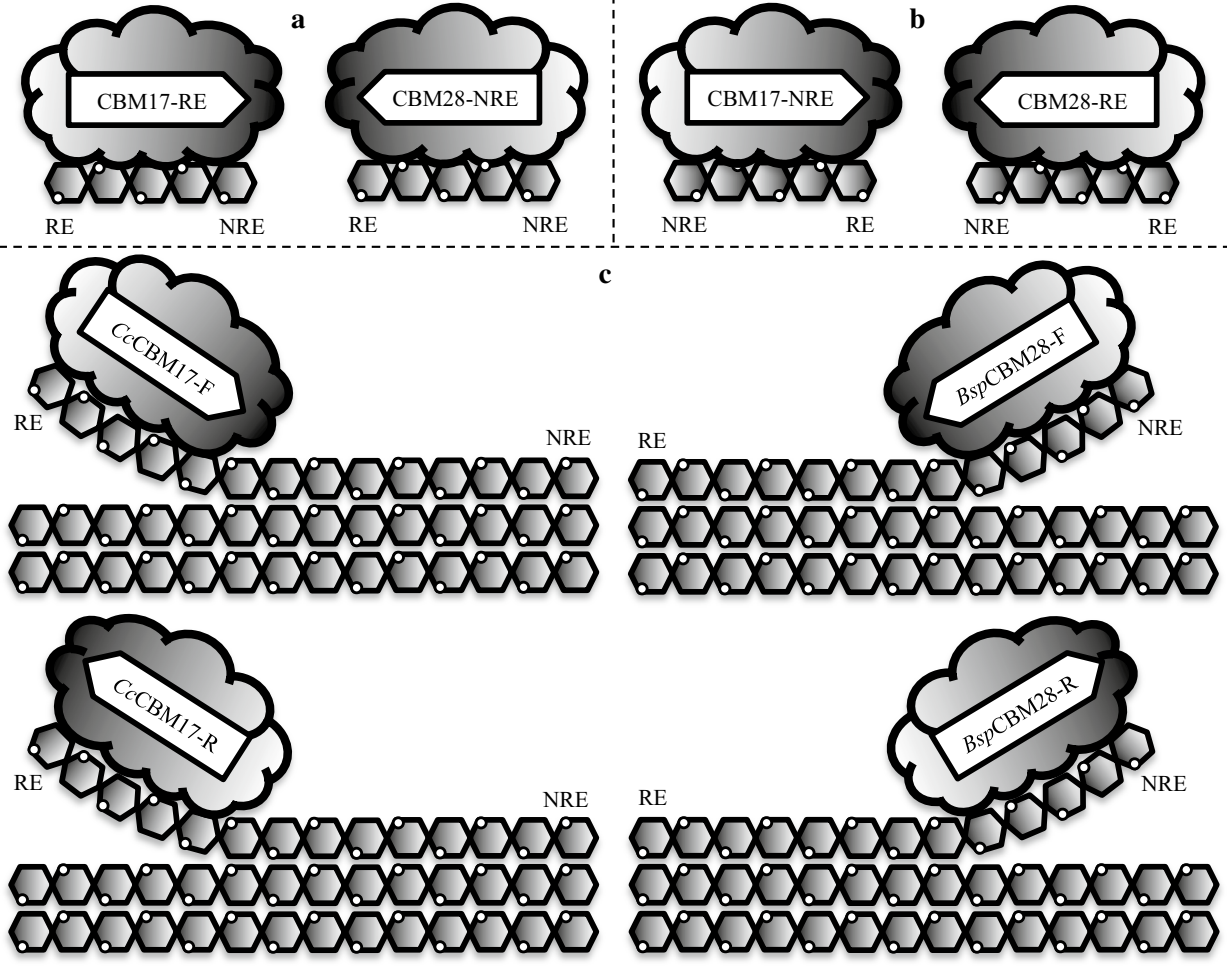
Cartoon illustration of the protein–carbohydrate complexes modeled in this study. CBMs from family 17 and 28 were modeled with cellopentaose bound in **a** the crystallographic structure orientation and **b** with the reducing end of the pyranose ring at the opposite end of the groove from the structural orientation. **c** CBMs were also bi-directionally bound with partially decrystallized cellulose Iβ microfibrils, representative of non-crystalline cellulose substrates. *RE* reducing end, *NRE* non-reducing end

To gain a molecular-level understanding of how these three families of Type B CBMs discriminate between binding soluble oligomeric and non-crystalline/amorphous substrates, we implemented a computational approach to describe the differences in binding behavior and affinities within and among the CBM families. From molecular dynamics (MD) simulations, we explored the role of binding site architecture. Free energy perturbation with Hamiltonian replica exchange MD (FEP/λ-REMD) and umbrella sampling MD was used to examine bi-directional ligand binding ability and apparent binding modes in non-crystalline substrate recognition. At each step of our study, we compared the computational results with available experimental data to assess the validity of our observations and to translate observations to practice. Two representative CBMs from each of the three CBM families, 4, 17, and 28, were selected to gain an understanding of the variations in protein–carbohydrate binding within and across the families. The selected representatives were *Cellulomonas fimi* CBM4–1 and CBM4–2 (*Cf*CBM4–1 and *Cf*CBM4–2), *Clostridium cellulovorans* CBM17 (*Cc*CBM17), *Bacillus* sp. *1139* CBM17 and CBM28 (*Bsp*CBM17 and *Bsp*CBM28), and *Clostridium josui* CBM28 (*Cj*CBM28). The representatives were selected on the basis that they are characterized as cellulose-specific Type B CBMs and are shown to have affinity for both oligomeric and non-crystalline cellulose. CBMs with available structural data were preferred to be able to convincingly apply computational techniques. Briefly, the protein–carbohydrate systems were modeled in the following configurations (Fig. 2): (A) CBMs with the cello-oligosaccharide bound in the orientation observed in the crystallographic structure, (B) CBMs with the oligosaccharide bound in the opposite direction of the structural orientation (i.e., with the reducing end of the sugar longitudinally rotated to the opposite end of the groove), and (C) CBMs bound with a partially decrystallized cellulose microfibril, representative of non-crystalline cellulose, in both the structural and reverse orientations. Additionally, each of the CBM representatives was modeled without a bound ligand for comparison, totaling 16 unique molecular models (Additional file 1: Table S1). The “Methods” section contains additional details on model construction to aid in understanding the “Results and discussion” section.

## Results and discussion

### Role of binding site architecture in substrate recognition

**Fig. 3:**
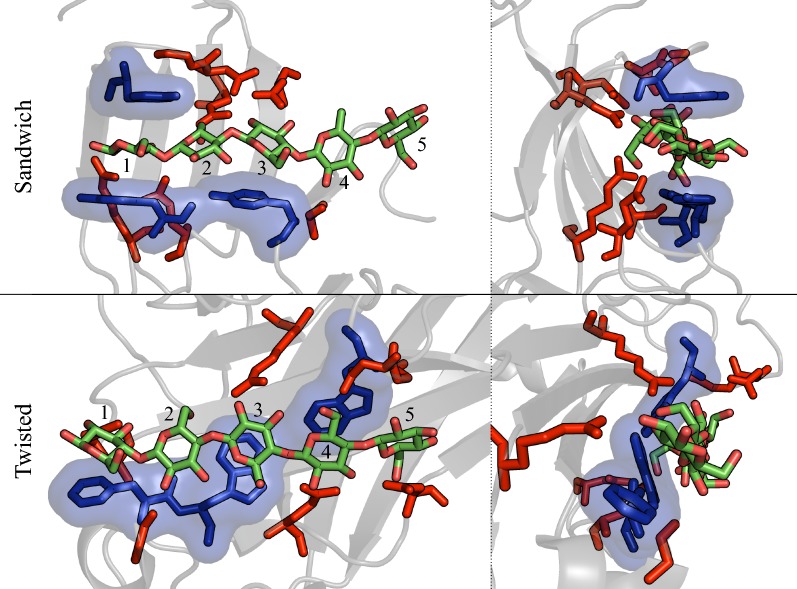
Differences in the two binding site architectures of family 4, 17, and 28 CBMs. Illustrated through hydrophobic interactions (dark blue sticks and transparent surface) and hydrogen bonding (red sticks) with the cellopentaose ligand (light green and red sticks). The front view (top left) and side view (top right) of the *Cf*CBM4-1 binding site with bound cellopentaose clearly show the sandwich platform and deep cleft with one-sided hydrogen bonding of the ligand. The front view (bottom left) and side (bottom right) of the *Cj*CBM28 binding site with bound cellopentaose show a twisted surface platform and shallow groove with hydrogen bonding partners available on both sides of ligand. The numbers on the cello-oligomeric ligand represents the binding subsite number

The three CBM families, 4, 17, and 28, share the same β-sandwich protein fold but exhibit key differences in binding site architectures/platforms. As they all belong to the Type B classification, the binding site generally conforms to either a cleft or groove capable of accommodating a single glycan chain. However, structural examination reveals the family 4 CBMs exhibit much deeper binding clefts relative to the more open grooves of family 17 and 28 CBMs, which we expect plays a critical role in substrate recognition mechanisms. Both *Cf*CBM4–1 and *Cf*CBM4–2 display aromatic residues lining the cleft and whose hydrophobic surfaces face each other to sandwich the substrate pyranose rings between them (Fig. 3). The oligomeric substrate is enveloped in a 4–5 Å-deep cleft with its pyranose ring perpendicular to the CBM surface [26]. Family 17 and 28 CBM binding grooves also display aromatic residues, although they are positioned side-by-side with their hydrophobic surfaces exposed to the solvent. Additionally, these aromatic residues are not exactly aligned in parallel planes, as in Type A CBMs, but, rather, comprise a shallow 1–2 Å groove with a ‘twisted’ polysaccharide-binding platform [24, 27].

The significance of individual hydrophobic aromatic residues and polar residues in both family 17 and 28 CBMs has been examined in prior experimental studies [24, 28, 29]; however, binding affinity studies suggest that, despite structural and sequence similarity, thermodynamic binding signatures are not always consistent within members of the same family [21, 27]. We have previously discussed the similarities and differences within the two family 4 CBMs for ligand binding dynamics and thermodynamic preference [25]. Here, we focus on comparing and contrasting oligomeric ligand binding modes and affinity across the two different binding platforms of CBM4s and CBM17 and 28s, ‘sandwich’ and ‘twisted’, respectively, as well as within and across the three Type B CBM families.

*Cf*CBM4–1-RE and *Cf*CBM4–2-RE (sandwich platforms), and *Cc*CBM17-RE and *Cj*CBM28-NRE (twisted platforms) are compared here, as experimental binding affinities and structures have been determined for each. Reported affinities for cellopentaose for each of the four CBMs, as well as structurally similar family members, are given in Table 1. Note that measurement techniques and experimental conditions vary by study making direct comparison challenging. For the same four CBM–cellopentaose systems, we calculated binding affinity using FEP/λ-REMD method under conditions similar to experiment, at 300 K and pH 7.0. The orientation of the ligands relative to the CBM binding cleft reflected the crystallographic configuration (Fig. 2a). We found that *Cf*CBM4–1 and *Cf*CBM4–2 exhibited affinities for cellopentaose of − 4.5 ± 1.3 kcal/mol [25] and − 5.4 ± 1.4 kcal/mol. *Cc*CBM17-RE and *Cj*CBM28-NRE exhibited affinities for cellopentaose of − 6.9 ± 0.9 kcal/mol and − 6.3 ± 0.7 kcal/mol, respectively. Detailed distribution of free energy components, including charge, dispersion, van der Waals, and restraining contributions (Table S2), and illustration of calculation convergence has been provided in Additional file 1: Fig. S3. While the trend is weak, the experimental and calculated affinities, combined, suggest moderately tighter cellopentaose binding may occur in some ‘twisted platform’ CBMs (i.e., families 17 and 28). These subtle thermodynamic preferences of different platforms are among the factors that play a role in building the recognition mechanisms targeted towards specific substrates, as we discuss below. Later in this study, we address our hypothesis that moderately tighter binding in the twisted platform is an evolutionary feature of family 17 and 28 CBMs that allows them to preferably recognize non-crystalline cellulose over cello-oligomers.

**Table 1:** Binding affinities of a given family 4, 17, or 28 CBM for cellopentaose

References	CBM	Method	Temperature (K)	pH	Buffer conditions	∆*G*° (kcal/mol)
Tomme et al. [23]	*Cf*CBM4-1	ITC	308	7.0	Pure water	− 5.23 ± 0.91
Tomme et al. [23]	*Cf*CBM4-1	ITC	308	7.0	50 mM potassium phosphate	− 6.09 ± 0.64
Tomme et al. [23]	*Cf*CBM4-1	ITC	308	7.0	50 mM potassium phosphate 1.0 M NaCl	− 6.21 ± 0.57
Brun et al. [58]	*Cf*CBM4-2	ITC	308	7.0	50 mM potassium phosphate 1.0 M NaCl	− 5.80 ± 0.005
Boraston et al. [22]	*Cc*CBM17	Fluorescence	298	7.5	25 mM Tris–HCl	− 6.72
Boraston et al. [22]	*Cc*CBM17	Fluorescence	298	7.5	25 mM Tris–HCl 0.5 M NaCl	− 6.93
Boraston et al. [22]	*Cc*CBM17	Fluorescence	298	7.5	25 mM Tris–HCl 1.0 M NaCl	− 7.05
Boraston et al. [22]	*Cc*CBM17	Fluorescence	298	7.5	25 mM Tris–HCl 2.0 M NaCl	− 7.24
Notenboom et al. [24]	*Cc*CBM17	ITC	298	7.0	50 mM potassium phosphate	− 5.80 ± 0.03
Boraston et al. [21]	*Bsp* CBM28	UV	298	7.0	25 mM Tris–HCl	− 5.81 ± 0.14
Boraston et al. [21]	*Bsp* CBM28	ITC	298	7.0	50 mM potassium phosphate	− 5.93 ± 0.003
Araki et al. [29]	*Cj*CBM17	ITC	293	7.0	100 mM potassium phosphate	− 7.7 ± 0.3
Araki et al. [29]	*Cj*CBM28	ITC	293	7.0	100 mM potassium phosphate	− 7.7 ± 0.6

The CBMs listed here include: *Cellulomonas fimi* CBM4-1 and CBM4-2 (*Cf*CBM4-1 and *Cf*CBM4-2), *Clostridium cellulovorans* CBM17 (*Cc*CBM17), *Bacillus* sp. *1139* CBM28 (*Bsp*CBM28), and *Clostridium josui* CBM28 (*Cj*CBM28). The methods for obtaining binding affinities in the referenced study are listed as either isothermal titration calorimetry (ITC), fluorescence emission scans, or ultraviolet (UV) difference spectra. Reported experimental conditions are as given

### MD simulations further differentiate binding platforms

**Fig. 4:**
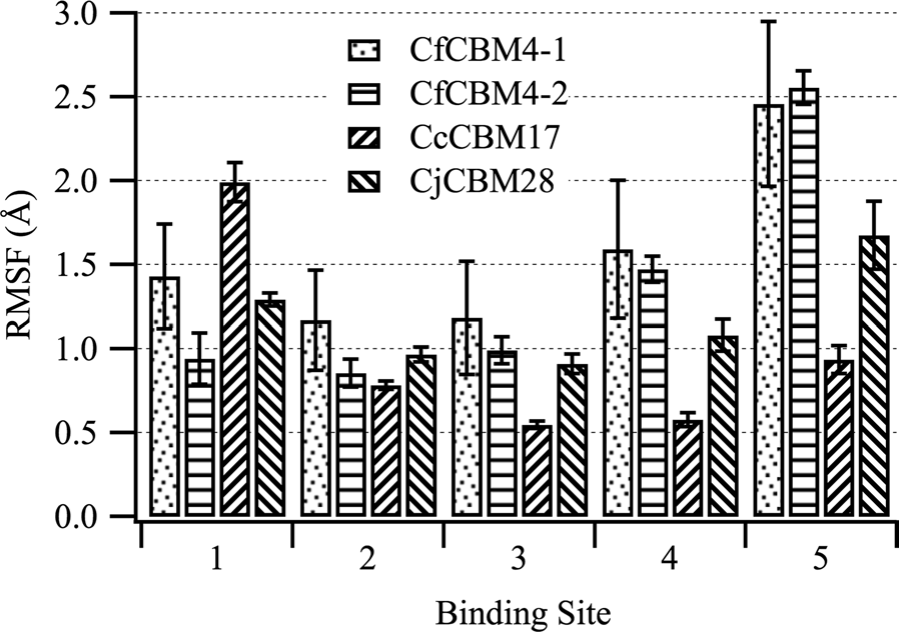
Root mean square fluctuation (RMSF) of cellopentaose. RMSF of the cellopentaose ligand from its average position in the clefts/grooves of representatives from family 4, 17, and 28 CBMs obtained from 250-ns MD simulation on a per-binding-subsite basis. Error was calculated from block averaging with block sizes of 2.5 ns

MD simulations provide additional insight into the binding free energy calculations, revealing that *Cc*CBM17-RE and *Cj*CBM28-NRE form more stable non-covalent interactions with the cellopentaose ligand than either family 4 CBM. From the 250-ns MD trajectories, we calculated the root mean square fluctuation (RMSF) of the ligand on a per-binding-subsite basis (Fig. 4); error was estimated by block averaging over 2.5 ns blocks. This value describes how much a given pyranose ring fluctuates from its average position over the course of a simulation. Collectively, as well as in nearly every binding site, the pyranose rings within the *Cf*CBM4–1 and *Cf*CBM4–2 binding cleft fluctuate more than that of either *Cc*CBM17-RE or *Cj*CBM28-NRE, indicating the latter two ligands form more stable protein–carbohydrate contacts. Moreover, the lower RMSF combined with the marginally higher binding affinity in the twisted platforms suggests that the unfavorable entropic penalty is compensated by enthalpic contributions, particularly hydrogen bonding.

There are three aromatic residues in the binding sites of the CBM4s and CBM28s, while CBM17s display only two, so the contribution to ligand binding from hydrophobic stacking interactions is not platform-dependent, varying by family. Rather, hydrogen bonding interactions appear to be a key determinant in affinity differences between the two binding site architectures. The average number of hydrogen bonds formed between a given pyranose ring with the side chains of the surrounding protein was determined using VMD; detailed analysis of hydrogen bonding over the course of the MD simulations identified the primary hydrogen bonding partners in all the CBM–oligomer interactions. The average number of hydrogen bonds formed per binding site was calculated from the 250-ns MD trajectories, where a hydrogen bond was defined as two polar atoms having a donor–acceptor distance of < 3.0 Å and a 20° cutoff angle. Table 2 shows the hydrogen bonding pairs from the calculations along with percent occupancy of each pair, where occupancy refers to the percent of the simulation during which the hydrogen bond was formed. While the CBMs with the same binding site architecture exhibit comparable hydrogen bonding, the total number of hydrogen bonds formed with the twisted platform was almost 100% higher than that of the sandwich platform. Total percent occupancy of 100% indicates that at any given time of simulation there is, on average, at least one hydrogen bond between the ligand and protein. Along the twisted platform, there are one or more additional hydrogen bonding partners, accounting for an additional 1–2 kcal/mol of binding free energy for the whole binding site [30, 31]. *Cc*CBM17-RE and *Cj*CBM28-NRE form more hydrogen bonds with cellopentaose than either *Cf*CBM4–1 and *Cf*CBM4–2, fitting with the conjecture that the loss of conformational entropy in ligand binding is compensated by enthalpic contributions to free energy. This difference in hydrogen bonding can be justified by analysis of the positioning of partner amino acid residues along the binding site. In the sandwich platform, where the ligand is approximately perpendicular to the protein surface, primary and secondary hydroxyl groups of only one edge of the cellopentaose chain contact the CBM and the other edge is exposed to solvent (Fig. 3). In contrast, the cellopentaose bound in the twisted platform hydrogen bonds with partners on both sides of the groove. In CBM4s, there are relatively few hydrogen bonding partners available at binding subsite 5, but in the case of *Cc*CBM17-RE and *Cj*CBM28-NRE, each binding subsite exhibits at least one residue capable of hydrogen bonding.

**Table 2:** Percent occupancy of each hydrogen bond formed between the pyranose ring at each binding site and the surrounding protein residue over the 250-ns simulation

	*Cf*CBM4–1			*Cf*CBM4–2		
	Donor	Acceptor	Occupancy (%)	Donor	Acceptor	Occupancy (%)
Sandwich platform	ARG75-Side	BGC1-Side	26.86	ARG81-Side	BGC1-Side	43.83
	BGC3-Side	ASN81-Side	25.70	BGC2-Side	GLN128-Side	40.02
	BGC4-Side	ALA18-Main	25.14	HSE132-Side	BGC2-Side	26.11
	BGC2-Side	TYR43-Main	16.61	BGC4-Side	LEU24-Main	7.83
	BGC2-Side	GLN124-Side	15.37	BGC4-Side	SER23-Side	6.96
	GLN128-Side	BGC1-Side	6.54	BGC3-Side	SER23-Side	5.24
	GLY82-Main	BGC2-Side	5.11	ASN56-Side	BGC3-Side	3.98
	GLN124-Side	BGC3-Side	2.23	SER23-Side	BGC3-Side	3.27
	BGC4-Side	ASN50-Side	1.90	SER23-Side	BGC4-Side	2.86
	ASN50-Side	BGC3-Side	1.57	GLN128-Side	BGC2-Side	1.47
	BGC2-Side	ASN81-Main	1.43	–	–	–
	BGC3-Side	GLN124-Side	1.11	–	–	–
	GLN124-Side	BGC2-Side	1.04			

	*Cc*CBM17-RE			*Cj*CBM28-NRE		
	Donor	Acceptor	Occupancy (%)	Donor	Acceptor	Occupancy (%)
Twisted platform	BGC3-Side	ASP54-Side	63.20	ARG83-Side	BGC3-Side	65.98
	BGC4-Side	GLN129-Side	59.43	ARG178-Side	BGC1-Side	54.69
	ARG92-Side	BGC2-Side	37.35	BGC4-Side	GLN131-Side	45.93
	BGC2-Side	ASP54-Side	27.80	BGC5-Side	ASP76-Side	26.08
	GLN129-Side	BGC3-Side	20.06	BGC2-Side	GLY127-Main	23.77
	ASN185-Side	BGC4-Side	15.67	GLY77-Main	BGC5-Side	7.54
	ASN137-Side	BGC1-Side	14.64	BGC5-Side	ASP135-Side	3.14
	ASN52-Side	BGC3-Side	9.25	BGC4-Side	ASP76-Side	2.68
	THR184-Side	BGC5-Side	2.64	GLN131-Side	BGC4-Side	1.67
	ARG92-Side	BGC1-Side	2.15	TRP129-Side	BGC4-Side	1.57
	BGC5-Side	THR184-Main	1.54	TRP78-Main	BGC5-Side	1.12
	BGC1-Side	ASN137-Side	1.08	–	–	–

Data are shown in decreasing order of occupancy. Pairs with occupancy lower than 1% are not shown. BGC is an acronym for β-d-glucose. A hydrogen bond was defined as two polar atoms having a donor–acceptor distance of < 3.0 Å and a 20° cutoff angle

**Fig. 5:**
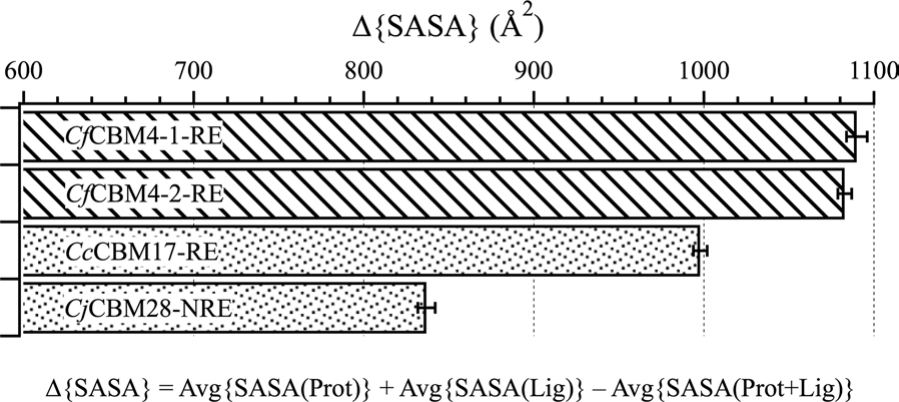
Average change in solvent accessible surface area (∆SASA) upon ligand binding. ΔSASA is calculated using VMD over the 250-ns MD simulation trajectories of each CBM–cellopentaose system to compare the difference between sandwich (lined pattern) and twisted (dotted pattern) platforms. The error bars represent the standard deviation of the mean

Average change in solvent accessible surface area (SASA) upon ligand binding (Fig. 5) reveals that the sandwich platform buried more solvent exposed surface area upon binding than the twisted platform, although the latter was more solvent exposed initially. The average change in SASA was calculated over 2500 frames of MD simulation, taking the difference between summation of average SASA of apo CBMs and average SASA of solvated cellopentaose, and average SASA of respective CBM–cellopentaose complexes (Fig. 5). The mean change in SASA is lower for twisted platform CBMs than sandwich platform CBMs, with less of a change in SASA observed for *Cj*CBM28-NRE than *Cc*CBM17-RE. The extra aromatic residue (Phe128) in the *Cj*CBM28 binding groove, being the most obvious difference within the twisted platforms of family 17 and 28 CBMs, appears to contribute to this difference, but it also suggests that having an aromatic residue may not always contribute to higher change in SASA as compared to sandwich platform CBMs that have three aromatic residues. Solvent-exposed residues along the twisted platforms do not appear to retain ordered water molecules proximal to the aromatic side chains when there is no bound ligand; upon ligand binding, additional water molecules were retained at the protein–carbohydrate interface, as hydroxyl groups of cello-oligosaccharides enable solvent reorganization [27]. The larger change in SASA for sandwich platform CBMs also reflects a conformational change upon ligand binding. As observed in the MD simulations, the deep cleft of the two family 4 CBMs narrows over time as it sandwiches the cello-oligomer and excludes water from the hydrophobic core of the protein. The twisted platform, on the other hand, does not appear to implement this sandwiching mechanism and may, rather, prefer sliding along the polysaccharide chain more freely.

Overall, MD simulation results, particularly through the observed hydrogen bonding patterns, suggest that the difference in cleft architecture (i.e., twisted vs. sandwich) contributes to differences in cellopentaose binding and, likely, protein–carbohydrate recognition mechanisms. It is tempting to suggest variations in molecular-level behavior, such as these, are a result of evolutionary necessity, where each binding site architecture is uniquely suited for targeting regional substrate features [13, 24].

### Extended binding sites of the twisted platform

**Fig. 6:**
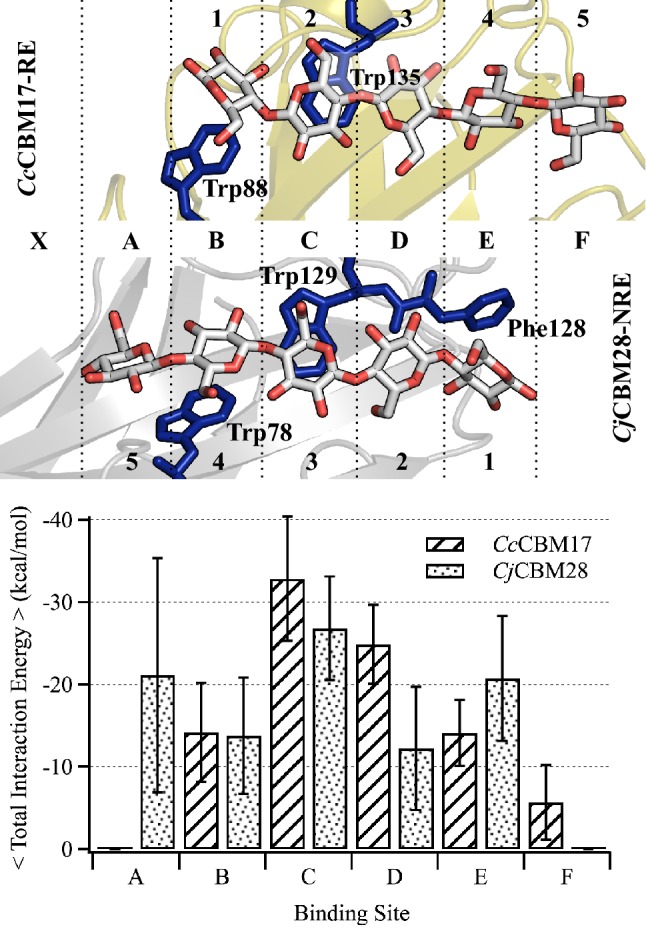
Alignment and comparison of the twisted platform binding sites. *Cc*CBM17-RE and *Cj*CBM28-NRE aligned with respect to the common pair of Trp residues (dark blue sticks) (top). The common naming of binding subsites used in this study (letters) is given between the alignment of the binding sites, and the original nomenclature (numbers) is given above and below the cartoon representations in the top panel. Average total interaction energy of the pyranose rings with the surrounding amino acid residues, on a per-subsite-basis, of *Cc*CBM17-RE and *Cj*CBM28-NRE calculated from the 250-ns trajectory (bottom). Error bars represent 1 standard deviation

To further differentiate oligomeric recognition mechanisms between *Cc*CBM17 and *Cj*CBM28, we compared ligand binding dynamics at each binding subsite (Fig. 6). Despite the apparent similarity in binding site architecture, the two CBMs feature cello-oligomers bound in opposite directions in their crystal structures (i.e., with the reducing end oriented at a different end of the groove when structurally aligned) [24, 27]. To enable comparison, the binding subsites of the two CBMs were structurally aligned, and a letter-based subsite nomenclature was invoked based on the two common solvent-exposed Trp residues (Fig. 6). The RMSF and hydrogen bonding evaluations reported above follow the numbered binding subsite nomenclature from crystal structure publications, as a cumulative comparison across the platforms. Alignment and renaming binding subsites (A to F), as previously implemented by Tsukimoto et al. [27], reveals that four common binding subsites (B, C, D, and E) are occupied by cellopentaose in *Cc*CBM17-RE (B to F) and *Cj*CBM28-NRE (A to E).

The average total interaction energy of protein with the cellopentaose ligand was determined from the 250-ns trajectory on a per-binding-subsite basis. The interaction energy distribution was very similar for CBM17 and CBM28 in the binding subsites B and C that reside along the hydrophobic face of a pair of Trp residues common to both CBMs (Fig. 6). Differences arise in the binding subsites as the extra aromatic residue in family 28 CBMs (Phe128 in *Cj*CBM28 and Tyr118 in *Bsp*CBM28) that can provide hydrophobic stacking interaction at binding site E. Nevertheless, we observe little difference at subsites D and E in the total interaction energy calculation that accounts for both van der Waals and electrostatic interactions. CBM17s and CBM28s are reported to bind oligomers as long as cellohexaose [21, 24], and it is apparent that, for CBM17s, the sixth subsite would be A, while for CBM28s, the sixth sugar can be accommodated in either F or X. As *Cc*CBM17 and *Cj*CBM28 are known to bind non-crystalline substrates as well, it is possible secondary binding subsites exist for chains even longer than cellohexaose. Accordingly, we docked cellohexaose with *Cj*CBM28 in two orientations, occupying subsites A to F and X to E, and conducted 100-ns MD simulations; these simulations showed that both subsite X and F functionally interact with the ligand, although X had a higher interaction than subsite F (Additional file 1: Fig. S4). Extended binding sites may play a critical role in the recognition of non-crystalline substrates.

### Bi-directional ligand binding extends to family 17 and 28 CBMs

In a previous study, we found that family 4 CBMs showed no thermodynamic preference towards a given longitudinal orientation of cello-oligomers (i.e., the oligomers can bind ‘bi-directionally’ with the reducing end of the chain at either end of the cleft); moreover, structural comparison of all 29 available (as of the publication date, June 2015) ligand-bound CBM structures exhibiting a β-sandwich fold revealed bi-directional binding occurs in many other β-sandwich CBM families [25]. We hypothesized bi-directional binding may be a feature β-sandwich CBMs developed as an evolutionary advantage, given that bi-directional binding could increase the probability of binding events up to twofold.

Here, we investigate the feasibility and mechanisms of the bi-directional binding phenomenon in family 17 and 28 CBMs. Although there are architectural differences in the binding sites of family 4 and family 17 and 28 CBMs, the approximate symmetry of cello-oligomeric ligands and redundancy of available hydrogen bonding partners in the cleft are the determining factors in bi-directionality, which is transferable over the architectures. To consider bi-directional binding within the twisted platform CBMs, we investigated the binding dynamics of four CBMs from families 17 and 28 (*Cc*CBM17, *Bsp*CBM17, *Bsp*CBM28, and *Cj*CBM28) with the cellopentaose ligand bound in both possible orientations in the binding grooves.

**Fig. 7:**
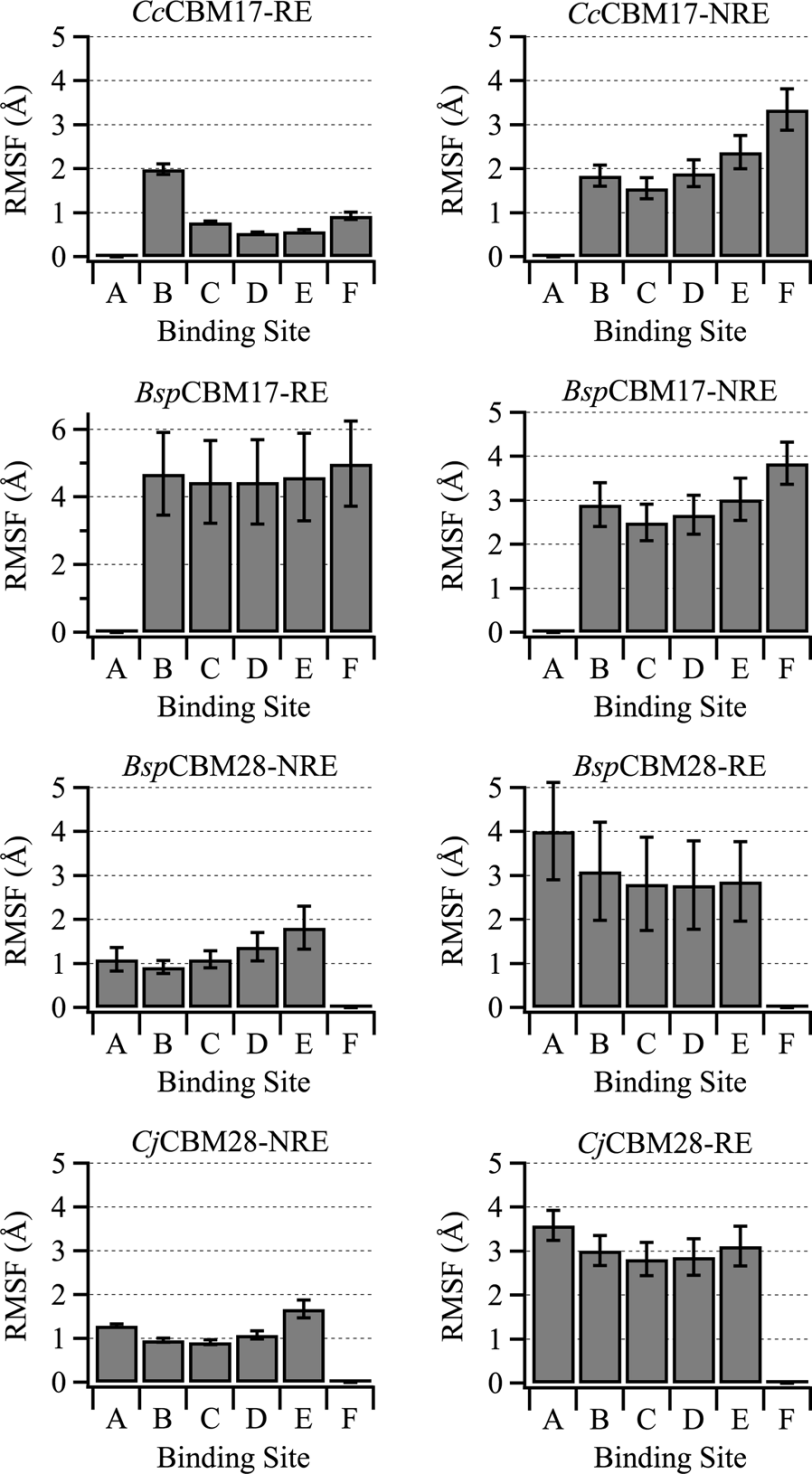
Root mean square fluctuation (RMSF) of the ligand. RMSF of the cellopentaose ligand from its average position over 250-ns trajectories. The RMSF was calculated on a per-binding-subsite basis for all eight family 17 and 28 CBM–cellopentaose systems. The error bars represent the standard deviation of block averaged RMSFs with block sizes of 2.5 ns each

In all eight simulation cases, the bound cellopentaose ligand maintained continuous interaction with the CBM binding surface over the entire 250-ns simulations, indicating that the binding sites of these family 17 and 28 CBMs can generally accommodate cello-oligomers bi-directionally. The RMSF of the ligand in the binding site provides a quantitative measure of stability of the interactions (Fig. 7), and while all four CBMs can accommodate the ligand bi-directionally, not all of them exhibit fully stabilized protein–ligand interactions. *Cc*CBM17-RE, *Bsp*CBM28-NRE, and *Cj*CBM28-NRE bind the cello-oligomer with relatively little fluctuation about the average (~ 1 Å). In the remaining five cases, though the cellopentaose ligands maintain contact with the CBM binding grooves, we observed sliding of the cellopentaose ligand along the binding site, which is reflected in the increased RMSF. We previously observed cellopentaose sliding within the CBM4 binding sites, however, the oligomers moved only a single subsite in either direction to rearrange the primary hydroxyl groups within the groove, as a result of the purposeful perturbation of ligand orientation [25]. The sliding observed in *Bsp*CBM17-RE, by two subsites or a cellobiose unit, maintains the primary and secondary hydroxyl group positions within a given subsite, which is suggestive of a functional mechanism rather than merely alleviation of steric hindrance. A cluster of snapshots (every 2.5 ns) from each simulation has been provided in Additional file 1 illustrating this phenomenon (Additional file 1: Fig. S5). In the case of *Bsp*CBM17-NRE, between 85 and 100 ns, the cellopentaose slides by one subsite, but an accompanying flip around the longitudinal axis maintains the original hydroxyl group orientation within the groove. Again, these results suggest the family 17 and 28 CBMs feature extended binding sites capable of binding cellohexaose or longer oligomers. Based on the alignment in Fig. 6, cellopentaose occupied subsites X and A in *Bsp*CBM17-RE and subsite A in *Bsp*CBM17-NRE (Additional file 1: Fig. S5).

These simulations support the hypothesis that the twisted platform of cellulose-specific CBMs also displays the characteristics of a binding site capable of bi-directionally binding cello-oligosaccharides. There exists a possibility that the uni-directional binding of cello-oligomers in these CBM crystal structures may have been favored by given conditions of crystallization, while in solution, these Type B CBMs have no thermodynamic preference for an orientation. However, experimental validation of this bi-directional binding phenomenon is desirable.

### Differentiation of high and low affinity binding sites on non-crystalline cellulose

Structural characterizations of many carbohydrate-active enzymes focus strictly on the interactions occurring in the carbohydrate binding site or catalytic active sites, while protein surface residues or secondary binding sites may be just as important to functionality [32]. Type B CBMs are reported to bind both cello-oligomers and non-crystalline/amorphous cellulose, covering a broad range of polymeric structural diversity and suggesting recognition processes may involve interactions beyond the primary binding site. Interestingly, CBMs from families 17 and 28 appear to bind non-crystalline cellulose with high and low binding affinities, as determined from isothermal titration calorimetry (ITC) data, and the two families do not compete with each other for carbohydrate binding sites [13, 33]. We further explore both the concept of bi-directional binding and the high/low binding affinity phenomena of family 17 and 28 CBMs on non-crystalline cellulose by modeling representative Type B CBMs bound with a model non-crystalline substrate in multiple orientations. At the nanoscale, we propose a partially decrystallized cellulose Iβ microfibril sufficiently represents the interaction of a CBM with non-crystalline cellulose, which retains a significant degree of crystallinity. Additionally, given our above insights into family 17 and 28 CBM members (i.e., that there is relatively little difference in binding dynamics of oligomers between members of the same family), we modeled only four representative CBM–microfibril systems: one CBM from each family attached to the decrystallized chain, or ‘whisker,’ in two possible orientations, forward and reverse (Fig. 2c). Details of this have been provided in the methods section below.

To explore the primary modes of Type B carbohydrate recognition with respect to non-crystalline cellulose, we used fully atomistic MD simulations based on previously validated cellulase–cellulose models [34]. All atomic interactions were unbiased except for the lower layer of the cellulose microfibril, which was harmonically restrained to prevent excessive fraying and further decrystallization. In all four cases, *Cc*CBM17-F, *Cc*CBM17-R, *Bsp*CBM28-F, and *Bsp*CBM28-R (Fig. 2c), the CBM–non-crystalline cellulose complexes stabilized in a global minimum state within the 100-ns MD simulations, illustrated by the rapid plateau in the protein backbone RMSD over time (Additional file 1: Fig. S6). Throughout the simulation, most CBMs bound all five pyranose moieties of the whisker along the twisted binding sites in the fully decrystallized state; in the case of *Bsp*CBM28-F, the fifth pyranose ring closest to the cellulose surface partially re-annealed into the microfibril, which is not unexpected [34]. Comparing RMSF of the CBM backbone when bound to either an oligomer or non-crystalline cellulose reveals that ligand binding stabilized the protein (lower RMSF); unbound CBM RMSFs exhibited larger fluctuations near binding site residues in both CBMs. Only *Bsp*CBM28-RE, bound with the rotated cellopentaose, showed large protein backbone fluctuations (Additional file 1: Fig. S7), resulting from ligand movement along the binding groove (Fig. 7) (Additional file 1: Fig. S7).

**Fig. 8:**
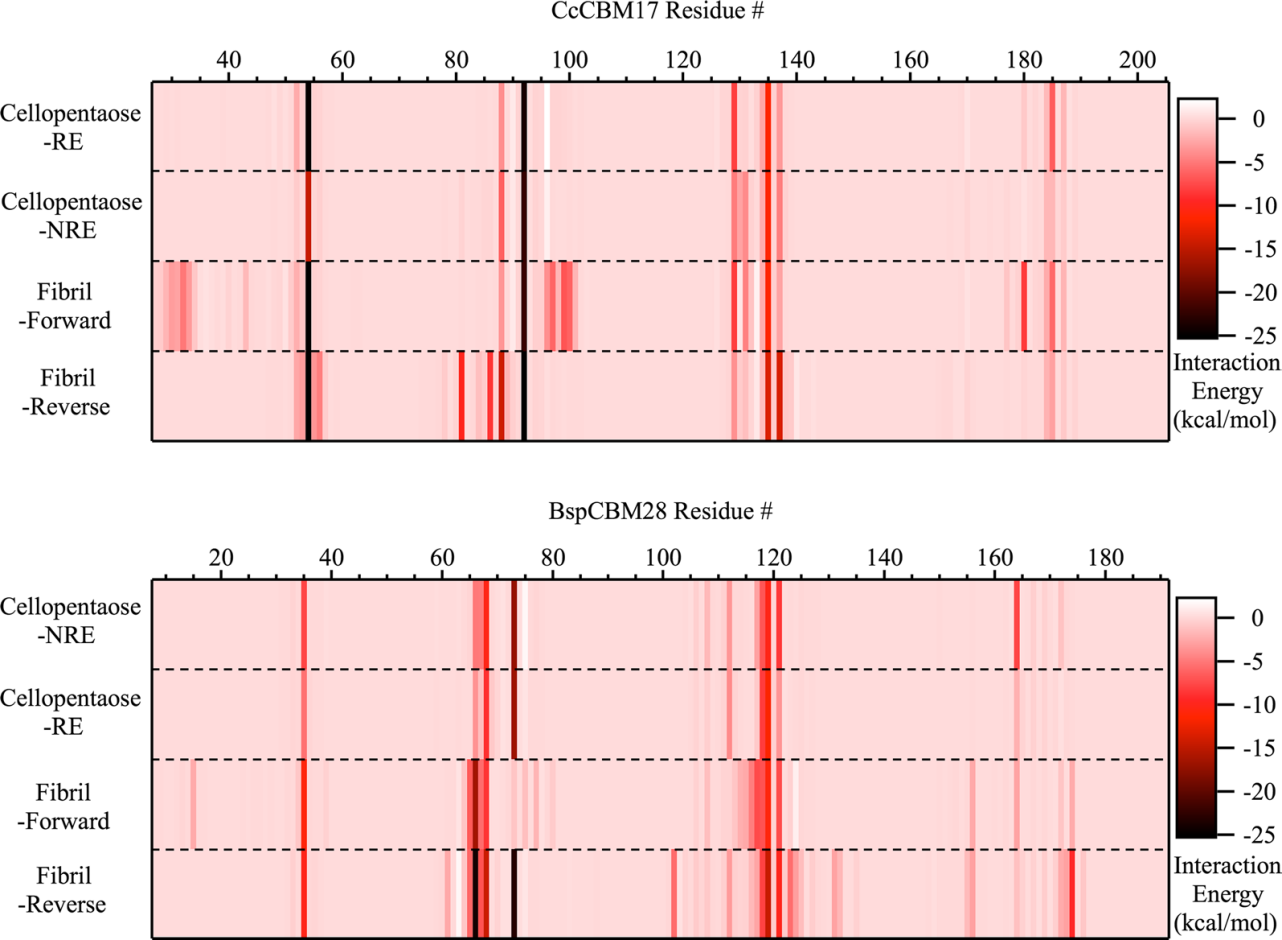
Total interaction energy between the substrate and each protein residue. Energies are averaged over the length of the MD simulations. The *Cc*CBM17 (top) and *Bsp*CBM28 (bottom) residue numbers are shown along the *x*-axis. The simulation case label is given at left, four cases for each family 17 and 28 CBM. The magnitude of the interaction energy between a given residue and the bound ligand, as indicated in the case name, is shown in white-red-black gradient. Favorable interactions are more negative and, thus, darker/black. In cellopentaose binding, ligand direction does not affect CBM–cellopentaose interactions, as redundant protein residues along the binding groove maintain an association with cellopentaose. In non-crystalline cellulose binding, the CBM protein surface interacts with the surrounding carbohydrate, in both forward and reverse orientations, to enhance binding affinity; the new protein–carbohydrate interactions are unique for each CBM and each direction

Comparing these four simulations and the oligomer-bound simulations above, we identified molecular-level factors contributing to substrate recognition in each family with respect to variation in substrate and orientation. The interaction energy of each CBM residue with the substrate was determined by averaging the calculation over trajectories, for each of the CBM–substrate systems (Fig. 8). For both family 17 and 28 CBMs, the average interaction of a given CBM residue with the substrate was independent of direction of cellopentaose ligand in the binding site, which is, again, consistent with bi-directional ligand binding. The hydrophobic-stacking aromatic residues and hydrogen bonding partners of the CBM–cellopentaose systems, as discussed above, produce substantial favorable interaction energies (< − 5 kcal/mol). These same residue–substrate interactions exist when the CBM is bound with non-crystalline cellulose. However, additional protein residues along the CBM surface also appear to be involved in binding non-crystalline cellulose (Fig. 8), as revealed from the rather significant new interactions formed in regions where the CBM–cellopentaose systems produce no such interactions.

While it is clear that protein surface residues play an auxiliary role in non-crystalline cellulose binding, each CBM and orientation relative to the cellulose surface results in a unique set of protein–substrate interactions to amplify non-crystalline cellulose binding affinity over oligomeric affinity. In the case of CcCBM17-F, two long peptide loops exterior to the binding groove, residues 30–35 and 95–106, interacted with cellulose as a result of their proximity to the cellulose surface in this ‘forward’ orientation. Most residues in these loops are polar residues, including Pro31, Lys32, Asp33, Asp96, Gln100, Ser101, Asn103, and Tyr105, and served to anchor the CBM over the microfibril through additional hydrogen bonding. In the case of *Cc*CBM17-R, Asp81, Asn86, and Asn137 produced new, large electrostatic interactions between the CBM and substrate. Also, aromatic residues like Trp88 produced more favorable interaction energies in the reverse orientation, while interacting loops in the forward orientation played no role at all. Similarly, for *Bsp*CBM28-F, the family 28 CBM lost hydrogen bond interactions between the ligand and Arg73 in the binding groove (subsite 3) and Gln112 (subsite 4); however, new hydrogen bond interactions with residues in loop 65–68 were formed. The *Bsp*CBM28-R orientation exhibited more consistent interaction patterns, with no loss of affinity contributors and formation of additional favorable interactions between cellulose and residues in loops 66–68 and 115–130. Ultimately, it seems each orientation of a given CBM relative to the cellulose surface produces a specific set of substrate interactions that enhance non-crystalline cellulose binding relative to binding of oligomers.

**Fig. 9:**
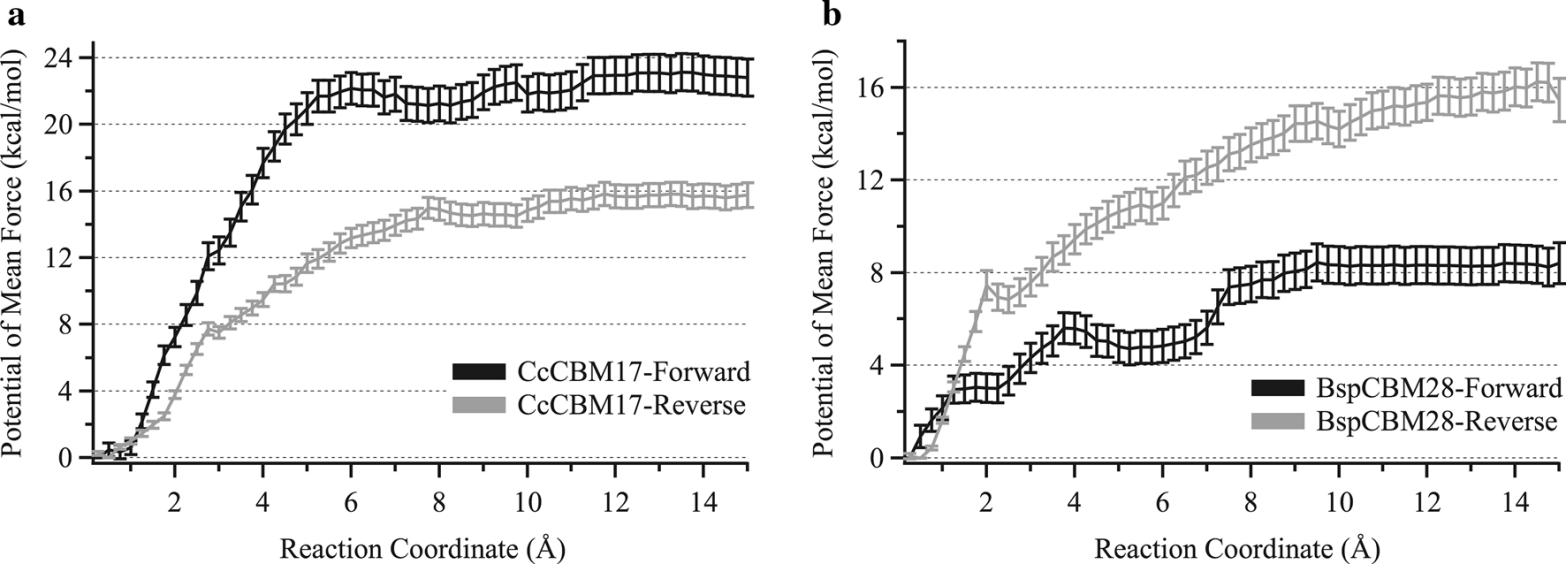
Potential of mean force (PMF) in uncoupling **a** CcCBM17 and **b** *Bsp*CBM28 from non-crystalline cellulose. Umbrella sampling MD was conducted over 30 0.5-Å windows using the projection of the distance vector on the *z*-axis as the reaction coordinate

To thermodynamically characterize the effects of orientation and substrate crystallinity on family 17 and 28 binding, we calculated binding affinities from the potential of mean force (PMF), or work required, to separate the CBMs from the non-crystalline cellulose substrate. We used umbrella sampling MD to disassociate the CBM from non-crystalline cellulose, pulling the CBMs away from the substrate perpendicularly. Sampling simulations were started from equilibrated 100-ns MD simulation snapshots of each CBM–non-crystalline cellulose complex. For all four cases, the corresponding PMFs indicate binding affinities are higher for non-crystalline cellulose than for oligomeric ligands in respective CBMs (Fig. 9); this result aligns with our hypothesis that the higher affinity binding sites described in experimental binding studies corresponds to CBM–non-crystalline cellulose binding and lower affinity binding sites correspond to CBM binding in oligomeric or highly decrystallized regions.

The PMF provides both a binding free energy and a quantitative view of the CBM dissociation process from a non-crystalline substrate (Fig. 9). The free energy of binding non-crystalline cellulose is determined from the difference between the free energy at the beginning (0 Å) and end (15 Å) of the reaction coordinate. For both *Cc*CBM17 and *Bsp*CBM28, the orientation of the CBM relative to the surface affects binding affinity, favoring the forward orientation in CBM17 and the reverse orientation with CBM28. Additionally, there is a significant difference in affinity between the two high-affinity orientations of each CBM family; *Cc*CBM17-F binds with the highest affinity, 23.0 ± 1.1 kcal/mol, and *Bsp*CBM28-R binds with an affinity equivalent to 15.9 ± 0.8 kcal/mol. Combined with the knowledge that these two CBM families do not competitively bind non-crystalline cellulose [13], our results suggest that CBMs from these two families are capable of recognizing cellulose binding sites based on binding orientations relative to the substrate. The difference between the affinity of CBM17 and CBM28 for non-crystalline cellulose may be correlated to the qualitative difference in the surface interactions that contribute to the affinity as well as fortuitous compatibility of CBM17s than CBM28s with proposed non-crystalline cellulose model. Decrystallized edge chain morphology could be one of the other cases of non-crystalline cellulose that are preferred by CBM28s over CBM17s because of the differences in the general surface topology around binding site of oligomers.

The model non-crystalline substrate simulated in this study represents a subset of cellulose morphologies that are very close to crystalline substrate, and the calculated free energies correspond to association constants as high as 10^12^ mol^−1^, which are not detectable by experimental methods such as ITC. The reported high-affinity cellulose binding sites for *Cc*CBM17 and *Bsp*CBM28 on regenerated cellulose, from ITC, were − 8.41 ± 0.32 and − 8.28 ± 0.35 kcal/mol, respectively [13] and while these values are much lower than those calculated from PMFs, it is plausible that the experimental affinities correspond to a range of other cellulose morphologies more amorphous in nature than the model non-crystalline substrate. Nevertheless, taken qualitatively together with the calculated and experimental values of cellopentaose binding to *Cc*CBM17 and *Bsp*CBM28, our results offer promising evidence that high and low affinity non-crystalline cellulose binding sites correspond to the degree of substrate crystallinity. In other words, these family 17 and 28 CBMs appear to bind cellulose with a higher degree of crystallinity with greater affinity than small, oligomeric substrates.

Finally, dissociation appears to occur in two separate events along the PMF profile (Fig. 9), with an initial exertion of work to decouple the CBM from the substrate surface and a final extrication of the polymeric chain from the CBM binding groove. The CBM bound with non-crystalline cellulose must initially overcome the strong electrostatic interactions and hydrogen bonds formed between the CBM protein surface and the cellulose surface. After the exterior of the CBM was free of the cellulose surface, the final amount of work required to dissociate the CBM was associated with overcoming both van der Waals interactions between with the aromatic residues and pyranose rings and several hydrogen bonds formed with the substrate along the length of the groove. Combined with our MD simulation results above, the increase in affinity observed in binding CcCBM17 and *Bsp*CBM28 with non-crystalline cellulose appears to be directly related to the additional protein–carbohydrate interactions mediated by residues exterior to the CBM binding groove.

## Conclusions

Through detailed analysis of protein–carbohydrate interactions, such as hydrogen bonding, and binding affinities for two different binding platforms observed within the same type of CBM, we found that binding site architecture appears to impact CBM functionality in recognizing carbohydrate substrates. Comparison of the twisted platforms in two different CBM families, 17 and 28, showed similarity in oligomeric ligand binding dynamics and established rationale towards possible extended binding sites. We have also addressed the questions raised by Boraston et al. in regards to mechanisms of Type B CBM–non-crystalline cellulose binding, expanding upon experimental observations identifying enthalpic interactions as dominant in non-crystalline substrate recognition by *Cc*CBM17 and *Bsp*CBM28 [35]. Specifically, we identified individual contributions to thermodynamic parameters, revealing that the gain in enthalpy in binding non-crystalline cellulose over oligomers results from direct contact of the CBM exterior with the cellulose substrate. We also provided insight into how family 17 and 28 CBMs uncompetitively bind non-crystalline cellulose, despite having very similar binding specificities and protein structure. The question of specifically assigning CBM–cellulose binding affinities to non-crystalline substrate binding sites remains, hinging on future experimental efforts to structurally characterize non-crystalline cellulose of increasingly amorphous nature. This study also provides the basis for our future investigations of glycoside hydrolases linked with tandem CBMs, as the two family 4 CBMs (*Cf*CBM4–1 and *Cf*CBM4–2) and the two *Bacillus* sp. *1139* family 17 and 28 CBMs (*Bsp*CBM17 and *Bsp*CBM28) are natural tandem constructs appended to β-1,4-endoglucanases. We anticipate the results toward understanding Type B CBM oligomeric and non-crystalline recognition mechanisms will advance our understanding of how protein–protein interactions and inter-module networking determines additive or cooperative binding in tandem systems and why organisms secret multi-modular enzymes with seemingly redundant CBM domains.

## Methods

### Modeling *protein–carbohydrate* complexes

Explicitly solvated models of each CBM were developed from Protein Data Bank (PDB) structures or via homology modeling. *Cf*CBM4–1 and *Cf*CBM4–2 models, in the apo and cellopentaose-bound states, were previously constructed [25]. *Cc*CBM17 was constructed from the 1J84 PDB structure, which features cellotetraose bound in the groove [24]. Similarly, *Bsp*CBM28 was constructed from the 1UWW PDB structure, having no bound ligand [36], and *Cj*CBM28 was constructed from the 3ACI PDB structure, featuring cellopentaose [27]. With no available crystal structure for *Bsp*CBM17, we used homology modeling, with *Cc*CBM17 as a template, to build the protein model [37, 38]; the two proteins are quite similar, having 55% sequence similarity and 70% structural similarity. For comparative purposes, we modeled the CBM-bound cello-oligomer as cellopentaose; an additional β-d-glucose residue was constructed near the end of the *Cc*CBM17 groove, and the cellopentaose ligand was docked with *Bsp*CBM17 and *Bsp*CBM28 by structural alignment with their homologous family member using the Dali pairwise alignment tool [39]. These four systems represent the oligomer-bound CBMs exhibiting the structural orientation, *Cc*CBM17-RE, *Bsp*CBM17-RE, *Bsp*CBM28-NRE, and *Cj*CBM28-NRE (Fig. 2a).

To investigate the bi-directional binding phenomenon in family 17 and 28 CBMs (Fig. 2b), we rotated the ligand from the structural orientation longitudinally along the ligand, as described for *Cf*CBM4–1 and *Cf*CBM4-2 [25]. Cellopentaose was docked in the opposite direction of that captured in the crystal structures by assuming the mean position of the pyranose ring heavy atoms must reside in approximately the same position regardless of direction. The approximate symmetry of the pyranose chair conformation enables this by merely exchanging the ring atom coordinates. CHARMM internal coordinate data was then used to establish the coordinates of the remaining sidechain atoms [40–42]. Extensive stepwise minimization of the ligand and the protein system was conducted before and after solvation to alleviate any deformation or bad contacts. These four systems, representing the “opposite” orientation, have been named *Cc*CBM17-NRE, *Bsp*CBM17-NRE, *Bsp*CBM28-RE, and *Cj*CBM28-RE for reference here.

We hypothesize high-affinity CBM-binding occurs when the CBM associates with amorphous or non-crystalline cellulose via partially decrystallized oligomeric chains decorating the top layers of degraded cellulose microfibrils (i.e., whiskers). Here, the partially decrystallized microfibril model used to represent amorphous/non-crystalline cellulose was adapted from the three-layer cellulose Iβ model used in previous cellulose decrystallization studies [43, 44]. The five-pyranose-long decrystallized segment was aligned with the cellopentaose from the equilibrated oligomeric systems described above using PyMOL (Additional file 1: Fig. S1). We docked two CBMs, a representative from both families 17 and 28 selected based on the availability of experimental affinity data for later comparison, in both ligand orientations such that we explore both possible interactions between these CBMs and non-crystalline cellulose. When aligned with each other or with *Cf*CBM4-1-RE (Additional file 1: Fig. S2), *Cc*CBM17 and *Bsp*CBM28, as obtained from crystal structures, appear to bind their cello-oligomeric ligands in opposite orientations, relative to the directionality of the core β-sheets. Assuming the structural orientations represent thermodynamically preferred recognition modes, we docked the *Cc*CBM17 on the cellulose reducing end and *Bsp*CBM28 on the cellulose non-reducing end and refer to them as *Cc*CBM17-F and *Bsp*CBM28-F (i.e., ‘forward binding mode’). A second set of systems were prepared with the CBMs in the ‘reverse binding mode,’ exploring both bi-directional binding and additional CBM-substrate recognition mechanisms. These ‘reverse’ systems are referred as *Cc*CBM17-R and *Bsp*CBM28-R (Fig. 2c). System construction was followed by extensive minimization and 1 ns of *NPT* equilibration to ensure the stability of the modeled protein–carbohydrate interaction and reduce solvation effects. During heating, equilibration, and production MD, the lower layer of the cellulose microfibril was restrained by applying harmonic restraints to the pyranose ring atoms; the CBMs and all other atoms of the systems were free of restraints. Protein alignment and ligand docking by alignment was carried out using PyMOL [45] and Dali pairwise comparison version 3.1 [39].

### MD simulation parameters and protocols

The CHARMM36 force-field with CMAP corrections was used to model all proteins [46, 47], and carbohydrates were modeled with the CHARMM36 carbohydrate force-field [40–42]. Water molecules were represented by the modified TIP3P force-field [48, 49]. Ions were modeled based on the force-field by Beglov and Roux [50]. Simulation setup and execution followed a procedure beginning with structure setup in CHARMM, vacuum energy minimization, explicit water solvation and charge neutralization with sodium ions, and extensive energy minimization [51]. The energy-minimized systems were then heated from 100 to 300 K over 20 ps and then simulated in the *NPT* ensemble for 500 ps to equilibrate the solution density (except where noted above). After equilibration, each apo CBM and CBM–cellopentaose system was simulated for 250 ns, and the CBM–microfibril systems were simulated in duplicate for 100 ns in the *NVT* ensemble using NAMD [52]. Additional parameter and protocol details have been provided in Additional file 1.

### Free energy calculations

We calculated the absolute free energies of binding cellopentaose to CBMs for all three families using an enhanced sampling free energy method, FEP/λ-REMD. FEP/λ-REMD is an enhanced sampling free energy methodology developed by Jiang, Hodoscek, and Roux [53], which we have previously implemented for protein–carbohydrate systems obtaining good agreement with experimental data [25, 54, 55]. For two different systems, the CBM–cellopentaose complex in solvent and solvated cellopentaose, the non-bonded interactions of cellopentaose with the rest of the system were systematically turned off to obtain the change in free energy. This free energy calculation protocol was implemented using a dedicated module for replica exchange in NAMD [52]. The non-covalent interaction between the CBM and cellopentaose was distributed into repulsive, dispersive, electrostatic, and restraining components over 128 replicas. All replicas were simulated simultaneously for 0.1-ns, and more than 20 such 0.1-ns consecutive windows were used to get 2-ns of sampling to ensure the convergence. The total change in free energy of binding was then calculated as the aggregate of ∆G_repu_, ∆G_disp_, ∆G_elec_, and ∆G_rstr_. The difference between the free energy of ‘disappearing’ cellopentaose from the CBM groove into vacuum and the solvation free energy of cellopentaose gives the absolute free energy of binding a solvated ligand to a solvated protein. Convergence of the free energy values was determined by Multistate Bennett Acceptance Ratio (MBAR) analysis method [56] and can depend on whether the model was prepared from crystal structure or homology model. Free energy calculations using models implementing ligand docking or homology modeling included additional restraining forces to improve convergence. For direct comparison, the FEP/λ-REMD calculations conducted here comply with the specifications outlined in our earlier study of family 4 CBMs [25]; accordingly, all methodological details are identical.

Umbrella sampling MD was used to determine the potential of mean force (PMF) of decoupling the CBM from the model non-crystalline surface into the solvent, from which we can estimate the free energy of binding. The distance between the projection of the center of mass of the CBM and the projection of center of mass of the lower layer of the cellulose microfibril on the *z*-axis served as the reaction coordinate. This distance was gradually increased by 15 Å in 0.5 Å increments, or 30 windows, until the non-bonded interaction between the protein and substrate no longer existed. The biasing force along the reaction coordinate was applied using collective variables during the 10-ns MD simulation of each window in NAMD [52]. To enable strictly perpendicular movement of the CBM relative to the microfibril surface, the distance between the same pair of projections on the *x*- and *y*-axes was restrained as a constant. The harmonic restraint on the ring atoms of the lower layer of the microfibril was maintained throughout sampling. A force constant of 10 kcal/mol was used to maintain the collective variables to their specified values. In *Cc*CBM17-F, the pyranose ring immediately prior to the decrystallized chain was harmonically restrained to the cellulose surface preventing further decrystallization as the CBM was pulled away. The last 5 ns of data was used in construction of the potential of mean force, discarding the first 5 ns as equilibration data. The reaction coordinates were normalized to represent the change in distance (i.e., 0 Å to 15 Å). The calculation of potential of mean force profile and error analysis was performed using MBAR [56].

## Additional file

**Additional file 1.** Details of molecular dynamics (MD) simulation methods and additional supporting figures and tables.

## Abbreviations

CBMs: carbohydrate binding modules
MD: molecular dynamics
FEP/λ-REMD: free energy perturbation with Hamiltonian replica exchange molecular dynamics
RMSF: root mean square fluctuation
SASA: solvent accessible surface area
ITC: isothermal titration calorimetry
PMF: potential of mean force
PDB: Protein Data Bank
MBAR: multistate Bennett acceptance ratio
