# Supplementary material for: Cellulose-specific Type B carbohydrate binding modules: understanding oligomeric and non-crystalline substrate recognition mechanisms
Source: Biotechnol Biofuels. 2018 Nov 30;11:319. doi: 10.1186/s13068-018-1321-7 (PMC6267901; doi:10.1186/s13068-018-1321-7)
Supplement: Supplementary file 1 — Additional file 1. Details of molecular dynamics (MD) simulation methods and additional supporting figures and tables. [file 13068_2018_1321_MOESM1_ESM.docx]

Additional file 1: Supporting data for "Cellulose-specific Type B carbohydrate binding modules: understanding oligomeric and non-crystalline substrate recognition mechanisms"

Abhishek A. Kognole^1^ and Christina M. Payne^1*^

^1^ Department of Chemical and Materials Engineering, University of Kentucky, 177 F Paul Anderson Tower, Lexington, KY 40506, USA.

^*^ Corresponding Author: Tel: +1-703-292-2895; e-mail: christy.payne@uky.edu

# Details for Molecular Dynamics (MD) Simulations

After acquiring the atomic coordinates from crystal structures (*Cc*CBM17 – 1J84, *Bsp*CBM28 – 1UWW, and *Cj*CBM28 – 3ACI) and homology modeling (*Bsp*CBM17), the pKa values of the CBMs’ titratable residues were determined at pH 7.0 using H++ web server ([Gordon et al. 2005](#_ENREF_3)). Visual inspection revealed additional residues to be protonated, including Asp200 in *Cc*CBM17, Asp72 in *Bsp*CBM17, Asp184 in *Bsp*CBM28, and Asp198 in *Cj*CBM28. Sixteen different molecular dynamics (MD) simulations were constructed using CHARMM ([Brooks et al. 2009](#_ENREF_1)). The systems, containing CBMs, crystallographic waters, calcium ions, and ligands (cellopentaose or microfibril), were constructed in vacuum and minimized for 1000 steps of Steepest Descent (SD) and 1000 steps of adopted basis Newton-Raphson (ABNR) with a tolerance of 0.01 for the average gradient. The vacuum-minimized systems were then solvated in explicit water, where the apo CBMs and CBMs bound with cellopentaose were solvated in a 70 Å × 70 Å × 70 Å cubic box (~35,000 atoms), and the CBMs bound with the cellulose microfibril were solvated in a 110 Å × 80 Å × 110 Å orthorhombic box. To neutralize the system charge, sodium or chloride ions were added by replacing random waters with the ions. The solvated systems were then subjected to extensive stepwise minimization: 2000 steps of SD with the protein and ligand fixed, 2000 steps of SD with only the protein heavy atoms fixed, and 10000 steps of SD and 10000 steps of ABNR (tolerance 0.01) with no restraints. The minimized systems were heated from 100 K to 300 K in 50 K increments over 20 ps, and then equilibrated for 500 ps in the *NPT* ensemble at 300 K and 1 atm. The Nosé-Hoover thermostat and barostat were used to control temperature and pressure in CHARMM ([Hoover 1985](#_ENREF_4); [Nose and Klein 1983](#_ENREF_6)). For the data collection (production) MD, in the *NVT* ensemble, the apo and oligomeric systems were simulated for 250 ns seconds, while the CBM-microfibril systems were simulated twice (independently) for 100 ns each. These simulations were carried out at 300 K using NAMD 2.10 ([Phillips et al. 2005](#_ENREF_7)). The Langevin thermostat was used to control temperature ([Schneider and Stoll 1978](#_ENREF_9)), and the SHAKE algorithm was used to fix the bond distances of all hydrogen atoms ([Ryckaert et al. 1977](#_ENREF_8)). Non-bonded interactions were truncated with a cutoff distance of 10 Å, a switching distance of 9 Å, and a non-bonded pair list distance of 12 Å. Long range electrostatics were described using the Particle Mesh Ewald (PME) method with 6th order b-spline, a Gaussian distribution of 0.320 Å, and a 1 Å grid spacing ([Essmann et al. 1995](#_ENREF_2)). The velocity Verlet multiple time-stepping integration scheme was used to evaluate non-bonded interactions every 1 time step, electrostatics every 3 time steps, and 6 time steps between atom reassignments. All simulations used a 2-fs time step.

**Figure S1**. Initial position of *Bsp*CBM28 in the forward orientation (after 500 ps of *NPT* equilibration) over the cellulose-Iβ microfibril with a middle chain of the top layer occupying the binding cleft of the CBM. The front view (left) and left-side view (right) illustrate the CBM (gray cartoon), its aromatic residues in the shallow binding cleft (blue sticks with transparent surface), and the cellulose microfibril (green sticks with red oxygens). A similar setup approach was used for the other three cases.

**Table S1.** List of all the MD simulations performed in this study with length of MD simulations and free energy calculation method.

| Group | CBM | Substrate | System Name | Simulation Time | Free Energy Calculation |
| --- | --- | --- | --- | --- | --- |
| Apo | *Cc*CBM17 | - | *Cc*CBM17 | 250 ns | - |
|  | *Bsp*CBM17 | - | *Bsp*CBM17 | 250 ns | - |
|  | *Bsp*CBM28 | - | *Bsp*CBM28 | 250 ns | - |
|  | *Cj*CBM28 | - | *Cj*CBM28 | 250 ns | - |
| A | *Cc*CBM17 | Cellopentaose | *Cc*CBM17-RE | 250 ns | FEP/λ-REMD |
|  | *Bsp*CBM17 | Cellopentaose | *Bsp*CBM17-RE | 250 ns | FEP/λ-REMD |
|  | *Bsp*CBM28 | Cellopentaose | *Bsp*CBM28-NRE | 250 ns | FEP/λ-REMD |
|  | *Cj*CBM28 | Cellopentaose | *Cj*CBM28-NRE | 250 ns | FEP/λ-REMD |
| B | *Cc*CBM17 | Cellopentaose | *Cc*CBM17-NRE | 250 ns | - |
|  | *Bsp*CBM17 | Cellopentaose | *Bsp*CBM17-NRE | 250 ns | - |
|  | *Bsp*CBM28 | Cellopentaose | *Bsp*CBM28-RE | 250 ns | - |
|  | *Cj*CBM28 | Cellopentaose | *Cj*CBM28-RE | 250 ns | - |
| C | *Cc*CBM17 | Cellulose microfibril | *Cc*CBM17-F | 100 ns + 100 ns | Umbrella Sampling |
|  | *Cc*CBM17 | Cellulose microfibril | *Cc*CBM17-R | 100 ns + 100 ns | Umbrella Sampling |
|  | *Bsp*CBM17 | Cellulose microfibril | *Bsp*CBM17-F | 100 ns + 100 ns | Umbrella Sampling |
|  | *Bsp*CBM17 | Cellulose microfibril | *Bsp*CBM17-R | 100 ns + 100 ns | Umbrella Sampling |

**Figure S2.** Structural alignment of *Cc*CBM17-RE and *Cj*CBM28-NRE with *Cf*CBM4-1-RE.

**Table S2.** Distribution of free energy components of cellopentaose (G5) binding to CBMs at 300K and pH 7. All values are in kcal/mol. Errors for ∆G_b_^°^ represent one standard deviation.

| System | ∆G_b_^°^ | ∆G_rep_ | ∆G_disp_ | ∆G_elec_ | ∆G_rstr_ |
| --- | --- | --- | --- | --- | --- |
| ^a^ G5 only | - | 68.04 ± 0.38 | -61.78 ± 0.01 | -66.25 ± 0.33 | - |
| ^a^ *Cf*CBM4-1 + G5 | -4.51 ±1.30 | 73.54 ± 0.19 | -78.87 ± 0.05 | -59.18 ± 0.15 | 0.29 |
| *Cf*CBM4-2 + G5 | -5.41 ±1.38 | 81.19 ± 0.33 | -81.01 ± 0.06 | -67.59 ± 0.17 | ^b^ 2.03 |
| *Cc*CBM17 + G5 | -6.94 ± 0.91 | 76.09 ± 0.19 | -77.27 ± 0.05 | -67.81 ± 0.18 | ^b^ 2.05 |
| *Cj*CBM28 + G5 | -6.26 ± 0.74 | 75.74 ± 0.19 | -72.55 ± 0.08 | -69.34 ± 0.17 | -0.11 |
| ^a^ Data obtained from [Kognole and Payne (2015](#_ENREF_5))  ^b^ Harmonic restraints were applied to rings atoms of ligand. | | | | | |


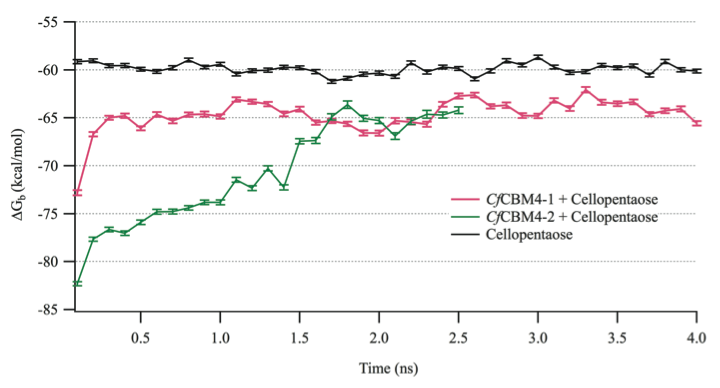

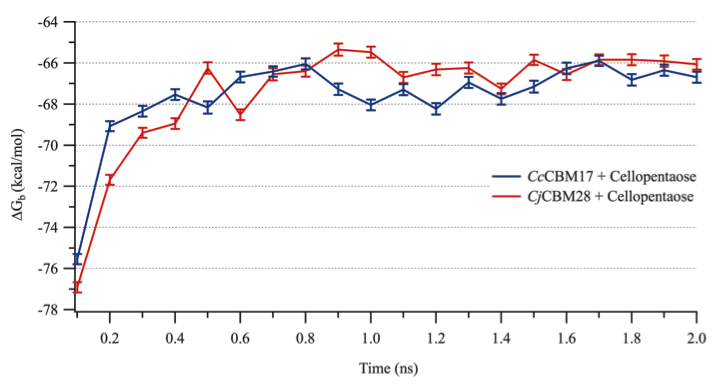


**Figure S3.** Convergence of the free energy calculations of cellopentaose binding to CBMs over consecutive windows of 0.1 ns using enhanced sampling method FEP/λ-REMD.

**Figure S4.** Average total interaction energy per binding subsite with the surrounding amino acid residues of *Cj*CBM28 for a cellohexaose chain of the microfibril occupying the cleft in two different ways, A to F (red) and X to E (blue). Values were calculated over the entire 100-ns trajectory. The error bars represent one standard deviation.


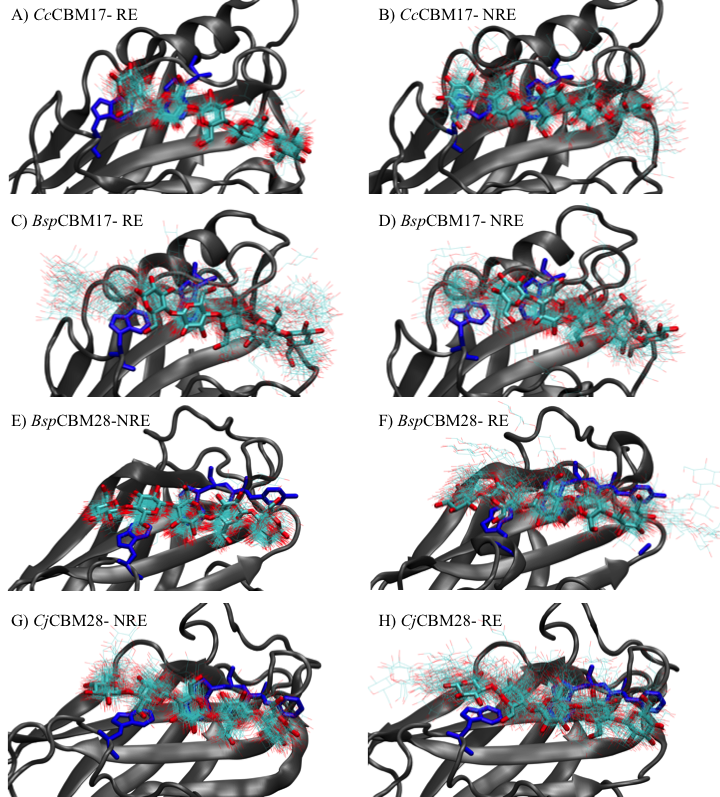


**Figure S5.** Snapshots of cellopentaose (lines) at every 2.5 ns in the binding site of each CBM (gray cartoon) over the 250-ns simulations. The position of cellopentaose at 0 ns is shown in thick cyan stick representation. The aromatic residues along the binding site are shown in dark blue stick representation.

**Figure S6.** RMSD of the CBM backbone bound to the model non-crystalline cellulose microfibril over 100 ns of MD simulation (10000 frames captured at every 0.01 ns). RMSD was determined with respect to the coordinates of each respective CBM at 0 ns.

**Figure S7.** Root mean square fluctuation (RMSF) of the backbone atoms of *Cc*CBM17 (top) and *Bsp*CBM28 (bottom) in each ligand occupancy state.

**References**

Brooks BR, Brooks CL, MacKerell AD, Nilsson L, Petrella RJ, Roux B, Won Y, Archontis G, Bartels C, Boresch S and others. 2009. CHARMM: The biomolecular simulation program. J. Comp. Chem. 30(10):1545-1614.

Essmann U, Perera L, Berkowitz ML, Darden T, Lee H, Pedersen LG. 1995. A smooth particle mesh Ewald method. J. Chem. Phys. 103(19):8577-8593.

Gordon JC, Myers JB, Folta T, Shoja V, Heath LS, Onufriev A. 2005. H++: a server for estimating pK(a)s and adding missing hydrogens to macromolecules. Nucleic Acids Res. 33:W368-W371.

Hoover WG. 1985. Canonical dynamics - equilibrium phase-space distributions. Phys. Rev. A 31(3):1695-1697.

Kognole AA, Payne CM. 2015. Cello-oligomer-binding dynamics and directionality in family 4 carbohydrate-binding modules. Glycobiology 25(10):1100-1111.

Nose S, Klein ML. 1983. Constant pressure molecular-dynamics for molecular-systems. Mol. Phys. 50(5):1055-1076.

Phillips JC, Braun R, Wang W, Gumbart J, Tajkhorshid E, Villa E, Chipot C, Skeel RD, Kale L, Schulten K. 2005. Scalable molecular dynamics with NAMD. J. Comp. Chem. 26(16):1781-1802.

Ryckaert JP, Ciccotti G, Berendsen HJC. 1977. Numerical-integration of cartesian equations of motion of a system with constraints - molecular-dynamics of N-alkanes. J. Comput. Phys. 23(3):327-341.

Schneider T, Stoll E. 1978. Molecular-dynamics study of a 3-dimensional one-component model for distortive phase-transitions. Phys. Rev. B 17(3):1302-1322.
